# Inability of myalgic encephalomyelitis/chronic fatigue syndrome patients to reproduce VO_2_peak indicates functional impairment

**DOI:** 10.1186/1479-5876-12-104

**Published:** 2014-04-23

**Authors:** Betsy A Keller, John Luke Pryor, Ludovic Giloteaux

**Affiliations:** 1 Department of Exercise & Sport Sciences, Ithaca College, School of Health Sciences & Human Performance, 318 Center for Health Sciences, Ithaca, NY 14850, USA; 2 Department of Kinesiology, University of Connecticut, Neag School of Education, 2095 Hillside Rd, Unit 1110, Storrs, CT 06269-1110, USA; 3 Department of Molecular Biology and Genetics, Cornell University, College of Agriculture and Life Sciences, 321 Biotechnology Building, Ithaca, NY 14853, USA

**Keywords:** Chronic fatigue syndrome, Functional impairment, Cardiopulmonary exercise test, Exercise intolerance, Post exertional malaise

## Abstract

**Background:**

Myalgic Encephalomyelitis/Chronic Fatigue Syndrome (ME/CFS) is a multi-system illness characterized, in part, by increased fatigue following minimal exertion, cognitive impairment, poor recovery to physical and other stressors, in addition to other symptoms. Unlike healthy subjects and other diseased populations who reproduce objective physiological measures during repeat cardiopulmonary exercise tests (CPETs), ME/CFS patients have been reported to fail to reproduce results in a second CPET performed one day after an initial CPET. If confirmed, a disparity between a first and second CPET could serve to identify individuals with ME/CFS, would be able to document their extent of disability, and could also provide a physiological basis for prescribing physical activity as well as a metric of functional impairment.

**Methods:**

22 subjects diagnosed with ME/CFS completed two repeat CPETs separated by 24 h. Measures of oxygen consumption (VO_2_), heart rate (HR), minute ventilation (Ve), workload (Work), and respiratory exchange ratio (RER) were made at maximal (peak) and ventilatory threshold (VT) intensities. Data were analyzed using ANOVA and Wilcoxon’s Signed-Rank Test (for RER).

**Results:**

ME/CFS patients showed significant decreases from CPET1 to CPET2 in VO_2_peak (13.8%), HRpeak (9 bpm), Ve peak (14.7%), and Work@peak (12.5%). Decreases in VT measures included VO_2_@VT (15.8%), Ve@VT (7.4%), and Work@VT (21.3%). Peak RER was high (≥1.1) and did not differ between tests, indicating maximum effort by participants during both CPETs. If data from only a single CPET test is used, a standard classification of functional impairment based on VO_2_peak or VO_2_@VT results in over-estimation of functional ability for 50% of ME/CFS participants in this study.

**Conclusion:**

ME/CFS participants were unable to reproduce most physiological measures at both maximal and ventilatory threshold intensities during a CPET performed 24 hours after a prior maximal exercise test. Our work confirms that repeated CPETs warrant consideration as a clinical indicator for diagnosing ME/CFS. Furthermore, if based on only one CPET, functional impairment classification will be mis-identified in many ME/CFS participants.

## Background

Myalgic encephalomyelitis/chronic fatigue syndrome (ME/CFS) is a multi-system illness that can lead to striking debilitation. Currently, diagnosis is based on a symptom profile. A hallmark symptom is referred to as “post-exertional malaise” (PEM), and encompasses disabling and persistent fatigue following exertion, usually accompanied by increases in other symptoms, including cognitive dysfunction [1]. Other common symptoms include, but are not limited to sleep disturbance, pain, and symptoms associated with autonomic dysfunction such as orthostatic intolerance, postural orthostatic tachycardia syndrome (POTS), light and sound sensitivity, and/or gastrointestinal distress. Fatigue in ME/CFS is not alleviated by bed rest and may be exacerbated by physical or cognitive activity, or other stressors [2]. While both sexes are afflicted, the incidence of ME/CFS is about 3–4 times greater in females [3].

Prevalence estimates for ME/CFS vary from 400,000 to 800,000 [3,4] to 1 to 4 million Americans who meet a case definition criteria for ME/CFS with fewer than 20% actually diagnosed [5]. Identifying an objective indicator of ME/CFS would be useful, particularly to accelerate a normally protracted path to diagnosis. Because post-exertional fatigue associated with ME/CFS contributes to physical activity intolerance, a measurement of maximal oxygen consumption (VO_2_peak) would be expected to indicate low aerobic capacity compared to normal values for age, sex, and activity level. In fact, measurement of aerobic capacity or VO_2_peak in ME/CFS patients is not standard clinical practice, although VO_2_peak has been used to characterize functional capacity in adults [6-13] and adolescents [14] with ME/CFS. Typically, patients and/or physicians may not seek assessment using cardiopulmonary exercise tests (CPET) to measure VO_2_peak until one has been physically inactive or low active for at least six months or longer. Not surprisingly, reported VO_2_peak values of adults with ME/CFS range from 30-91% of healthy controls or predicted values for age and sex [7-14] and 86-90% of healthy controls in adolescents with CFS [14]. While low, these values are generally consistent with physical deconditioning and are often not considered to be clinically relevant. In other words, a low VO_2_peak from a single CPET reveals low functional capacity, but does not allow the conclusion that the subject responds abnormally to exercise. However, ME/CFS patients report that post-exertional fatigue is not alleviated by rest and sometimes persists for days or weeks following an exercise challenge [1]. Post-exertional malaise, or the exacerbation of symptoms following an increase in a ME/CFS patient’s typical level of activity, dramatically impacts the ability to carry out both physical and cognitive activities of daily living. This major symptom of ME/CFS is included in the most commonly used clinical [1,15] and research [16] case definitions.

As pointed out by Snell et al. [17], the predominant ME/CFS case definitions fail to operationally define, or provide guidance to assess responses to exertion. To learn more about the impact of physical activity on subsequent physical function, a two-test maximum effort CPET protocol has been used to assess the ability of ME/CFS patients to reproduce VO_2_peak 24 hours after an initial CPET [13,17,18]. It is well documented that VO_2_peak is highly reliable (test-retest difference ≤ 7%) [19-21] and reproducible (r ≥ 0.95-0.99) [20,22,23] in healthy active [21] and inactive adults [19,23], children [24] and many patient populations [25-30]. Thus, failure of ME/CFS patients to reproduce VO_2_peak within the well-established normative variation of ≤ 7% would indicate an underlying pathophysiology, and could provide a metric of the effects of PEM on physical activity tolerance and physical function. To date, studies of physical activity tolerance in ME/CFS using the two-CPET protocol are few [13,17,18], but indicate an impaired ability of ME/CFS patients to reproduce CPET results. For example, studies revealed an inability of ME/CFS to reproduce VO_2_peak [13,18], VO_2_ at ventilatory threshold [18] or work at peak and/or ventilatory threshold intensities [17] within normative variation. However, collectively, these studies have yet to provide consensus on a physiological indicator(s) of impaired metabolic response to exercise. While these studies reveal obvious physiological anomalies in the ME/CFS response to exercise stress, limited sample size [13,18] and contrary results [17] call for additional evidence to more clearly elucidate the abnormal exercise responses in ME/CFS. More information about the response of ME/CFS patients to exercise will help to further clarify their abnormal physiology and objectively document functional impairment. Based on the previous two-day CPET studies, we hypothesized that ME/CFS would be unable to reproduce normally physiological indices during a second CPET performed 24 hours following an initial CPET. Therefore, the purpose of this study was to assess the reproducibility of VO_2_peak in ME/CFS patients, and secondly, to examine if a post-exertional measure of VO_2_peak would change the classification of functional impairment using a standard classification scheme.

## Methods

### Participants

Participants were 22 patients ill with ME/CFS for greater than 6 months and were diagnosed by referring physicians based on Fukuda et al. [15]. Each completed a health/medical history form and cardiovascular screening index to ascertain health status for inclusion in the study. Patients were excluded whose cardiovascular status was determined to be high-risk based on published guidelines for cardiovascular disease risk assessment [31]. Seventeen females (44.8 ± 11.37 yrs) and five males (39.8 ± 13.92 yrs) were free from co-morbidities or orthopedic limitations that would affect ability to complete a maximum cycle test. The sample distribution was proportionate with the reported incidence of ME/CFS by sex (72% females; [3]). The institutional review boards of Ithaca College and Cornell University approved the study, and written informed consent was obtained from participants.

### Procedures

Participants were instructed to abstain prior to the exercise tests from, 1) consuming food or smoking for 2 h, 2) caffeine or alcohol for 4 h, and 3) exercise for 24 h, and to use prescribed medications as usual on both test days. Next, they completed two CPETs (test 1, test 2) on a cycle ergometer separated by 23–24 hours.

Procedures for CPET began with pre-test resting measures of 12-lead ECG (Quinton Q4500 ECG, Quinton Instrument Co., Bothell, WA), blood pressure (BP) and heart rate (HR) that were made following five minutes of supine rest. The CPET began with 3 minutes of seated rest followed by cycling that began at 0 Watt and increased 25 Watts every two minutes until volitional exhaustion, a request to stop, or termination criteria were met [31]. Pedal cadence was 60–70 rpm on an electro-magnetically braked cycle ergometer (Lode Corival, Lode B.V., Groningen-Holland Medical Technology, Netherlands). Rating of perceived exertion (RPE; 6–20 scale [32]) was collected during the last 15 seconds of each workload, BP during the last minute of each workload, and ECG during each minute of the test. BP and ECG were monitored during recovery until the participant was within 20 bpm of pre-test resting HR and 10 mmHg of resting BP. Cycle seat height was positioned to approximately 175° of knee extension, and the same seat height was used for both tests. Expired gases were collected breath-by-breath through a two-way breathing valve, and analyzed using open circuit spirometry. The metabolic measurement system (PARVO Medics True Max 2400 metabolic measurement system, Salt Lake City, UT) was calibrated before each test with ambient air, standard gases of known concentrations and a 3-L calibration syringe. Ventilatory threshold (VT) is an analog of anaerobic threshold, and was identified from expired gases using the V-Slope [33] algorithm in the metabolic measurement system software. Ventilatory or anaerobic threshold is the exercise intensity at which metabolism transitions toward increased anaerobic energy production. The same trained investigator performed visual assessment and confirmation of the algorithm-derived VT. Testing took place in a controlled environment with temperature range of 20-24°C and 15-60% relative humidity.

### Statistical analysis

Physiological and work variables at maximum and VT intensities were compared between CPETs using repeated measures ANOVA for VO_2_, heart rate, Work, and minute ventilation (Ve), as well as for variables derived from these measures. Maximal respiratory exchange ratio (RERpeak) was compared between CPETs using a non-parametric Wilcoxon’s Signed-Rank Test. Statistical significance was P < 0.05. Analyses were completed with SPSS (version 20, Armonk, NY:IBM Corp.).

## Results

Participant ages ranged from 25 to 57 yrs with a mean of 43.7 ± 11.82 yrs and a body-mass index (BMI) of 27.4 ± 6.59 (Table 1), characterizing this cohort as overweight. In test 1, participants exhibited an average VO_2_peak of 21.9 ml^.^kg^.^min^-1^, which is low, and only 77.1% of the predicted VO_2_peak for age/sex-matched sedentary norms [23]. Physical characteristics and aerobic capacity of participants in this study were comparable to the 51 female ME/CFS participants studied by Snell et al. [17], whose age = 46.29 yrs, BMI = 25.96 and VO_2_peak (test 1) = 21.51 ml^.^kg^.^min^-1^. Likewise, Vermeulen et al. [18] studied 15 female ME/CFS patients who were younger (35.5 yrs) with normal weight status (BMI = 23.1), but had a similarly low aerobic capacity (test 1 VO_2_peak = 22.3 ml^.^kg^.^min^-1^) compared to the participants of the present study.

**Table 1:** Physical characteristics and aerobic capacity of participants, N = 22 (mean ± SD)

Age (y)	43.7 (11.82)
Height (cm)	167.3 (10.19)
Weight (kg)	76.8 (20.28)
BMI (kg^.^M^-2^)	27.4 (6.59)
VO_2_peak - Test 1 (ml^.^kg^-1.^min^-1^)	21.9 (4.75)
METs@peak (1 MET = 3.5 ml^.^kg^-1.^min^-1^)	6.26 (1.36)
%predVO_2_peak* - Test 1	77.1% (22.22)

*%predicted VO_2_peak for sedentary subjects from Bruce et al. [23].

**Figure 1:**
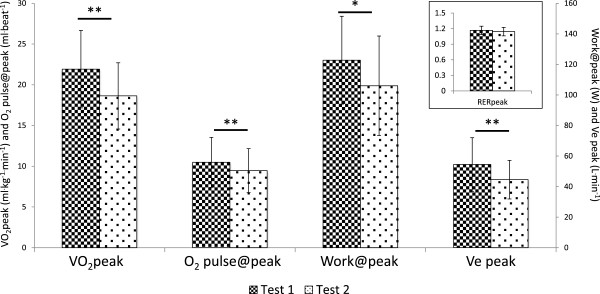
**Changes in physiological and work variables from Test 1 to Test 2 at maximal intensity.** Inset: Non-significant test differences for maximal respiratory exchange ratio showed that subjects achieved consistently high RER (>1.1) for Test 1 and Test 2, with maximum efforts on both tests (P = .157). Statistical significance is shown above bars with **P < 0.01 and *P < 0.05.

**Figure 2:**
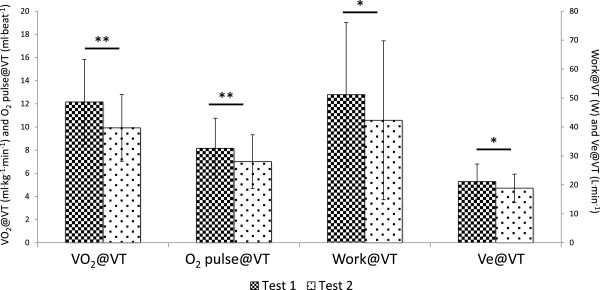
**Changes in physiological and work variables from Test 1 to Test 2 at ventilatory threshold.** Statistical significance is shown above bars with **P < 0.01 and *P < 0.05.

**Figure 3:**
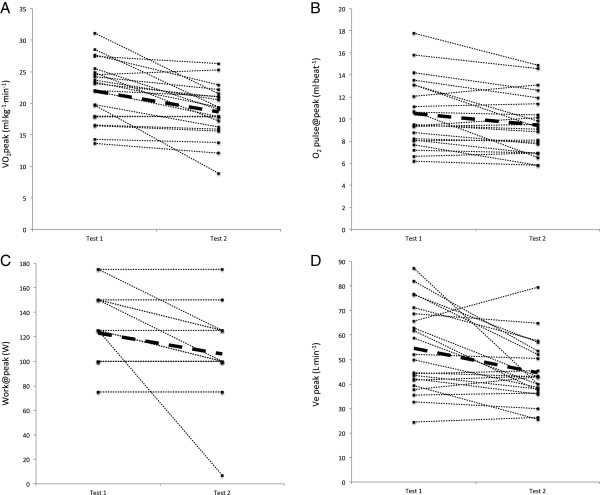
**Individual changes in peak measures of VO2 (A), O2pulse (B), work (C) and Ve (D) from Test 1 to Test 2.** Subjects’ whose VO_2_peak did not decrease during Test 2 showed a decrease in VO_2_@VT.

**Figure 4:**
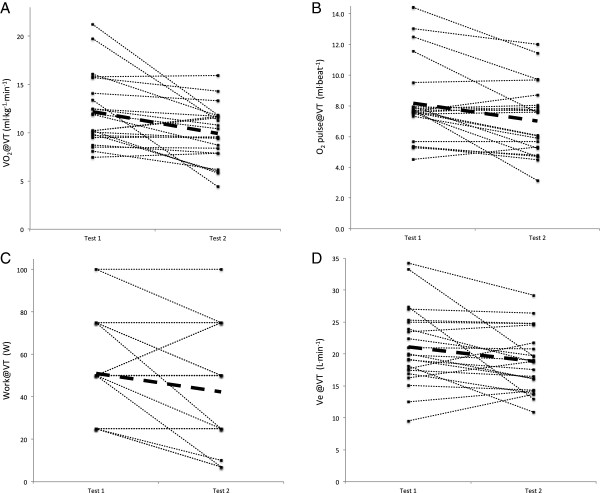
**Individual changes in ventilatory threshold measures of VO2 (A), O2pulse (B), work (C) and Ve (D) from Test 1 to Test 2.** Subjects’ whose VO_2_@VT did not decrease during Test 2 showed a decrease in VO_2_peak.

Test-retest changes in physiological and work variables appear in Table 2 and Figures 1 and 2. We detected significant differences in most parameters that were measured. Significant percent changes from test 1 to test 2 for measures at peak exercise decreased between 8.8 and 16.1%, and measures at ventilatory threshold decreased between 7.4 and 21.3%. Changes in physiological measures from test 1 to test 2 for individual subjects appear in Figures 3 and 4 for peak and ventilatory threshold intensities, respectively.

**Table 2:** Physiological and work variables for Tests 1 and 2 at peak and ventilatory threshold (VT) intensities, N = 22 (mean ± SD)

**Peak exercise**	**Test 1**	**Test 2**	%diff*	**P**
VO_2_peak (ml^.^kg^-1.^min^-1^)	21.9 (4.75)	18.6 (4.06)	-13.8%	.000^¶^
%predVO_2_peak^‡^	77.1% (20.22)	65.2% (15.74)	---	.000^¶^
HRpeak (bpm)	159.4 (21.10)	150.0 (23.05)	-5.9%	.001^¶^
%predHRpeak^†^	91.0% (10.75)	85.2% (11.93)	---	.002^¶^
Work@peak (W)	122.7 (28.77)	105.7 (33.57)	-12.5%	.012^||^
Ve peak (L ^.^min^-1^)	54.5 (17.56)	44.6 (12.63)	-14.7%	.003^¶^
VCO_2_peak (L ^.^min^-1^)	1.91 (.477)	1.58 (.464)	-16.1%	.000^¶^
O_2_ pulse@peak (ml^.^beat^-1^)	10.48 (3.068)	9.46 (2.697)	-8.8%	.003^¶^
%predVO_2_peak^‡^	77.1% (20.22)	65.2% (15.74)	---	.000^¶^
RERpeak	1.17 (0.079)	1.14 (0.081)	-1.9%	.157
**Ventilatory threshold**				
VO_2_@VT (ml^.^kg^-1.^min^-1^)	12.2 (3.68)	9.9 (2.89)	-15.8%	.003^¶^
HR@VT (bpm)	113.5 (21.78)	107.9 (20.61)	-4.9%	.086
Work@VT (W)	51.4 (24.97)	41.4 (28.8)	-21.3%	.030^||^
Ve@VT (L^.^min^-1^)	21.2 (6.07)	18.8 (4.86)	-7.4%	.035^||^
VCO_2_@VT (L^.^min^-1^)	0.86 (.343)	0.72 (.265)	-11.3%	.014^||^
O_2_ pulse@VT (ml^.^beat^-1^)	8.15 (2.603)	7.00 (2.323)	-12.6%	.003^¶^

*A negative %diff value indicates a decrease from Test 1 to Test 2.

^†^Percent of age-predicted maximum heart rate achieved.

^‡^% predicted VO_2_peak for sedentary subjects from Bruce et al. [23].

^||^Statistically significant difference between Test 1 and Test 2 at P < 0.05.

^¶^Statistically significant difference between Test 1 and Test 2 at P < 0.01.

Participants must achieve valid indicators of maximal effort to ensure that peak exercise data reflect strong effort. Maximal effort is indicated by RER ≥1.1 [31]. The use of RER as a metric of effort during CPET is consistent with the report by the American Heart Association [22] that maximum RER during exercise is the most accurate and reliable indicator of effort. In this cohort, RER at maximal effort (RERpeak) was high (≥1.1) and did not differ between tests, indicating that ME/CFS participant effort was very strong during both CPETs.

All measures at maximal intensity decreased in test 2, including VO_2_, HR, Work, minute ventilation and CO_2_ production (VCO_2_). One variable, O_2_ pulse, a surrogate for cardiac output (O_2_ pulse@peak) was derived as VO_2_/HR. Like both VO_2_ and HR at peak exercise, O_2_ pulse also decreased indicating reduced oxygen delivery in test 2. Similarly, at ventilatory threshold intensity, all variables, except HR, decreased during test 2. A decrease in HR@VT of almost 6 bpm approached but did not achieve statistical significance (P = 0.086). The larger coefficient of variation for HR@VT (~19% for tests 1 & 2) compared to HR at maximal effort (13% for test 1, 15% for test 2) likely contributed to the lack of statistical significance. Collectively, these data indicate that ME/CFS participants were unable to reproduce most physiological measures at both maximal and ventilatory threshold intensities during test 2, despite exercising to maximal effort during both tests. Examination of individual changes from test 1 to test 2 in Figure 3 reveal that VO_2_peak decreased in most patients and did not change in the remaining patients. Patients whose VO_2_peak did not change instead demonstrated a decrease in VO_2_@VT shown in Figure 4. Thus, all patients demonstrated clinically significant decreases in either VO_2_peak and/or VO_2_@VT that exceed normative values for test-retest variability.

Classifying functional impairment, based on VO_2_peak or VO_2_ at ventilatory threshold, could differ for ME/CFS patients due to a decrease in CPET measures at the time of the second CPET (Table 3). Using the established classification system of Weber & Janicki [34], the functional impairment classification based on VO_2_peak decreased in eight participants (37%) due to the change in VO_2_peak from test 1 to test 2. When using VO_2_ at ventilatory threshold to classify functional impairment, the classification decreased in 12 participants (55%). Overall, classification of functional impairment worsened in 50% of the ME/CFS cohort due to post-exertional decrements in VO_2_peak and/or VO_2_ at VT. Using only a single CPET, 13 of 22 were classified as “A” (little to no impairment) and eight were classified as “B” (mild-moderate impairment), which would typically be attributed to physical deconditioning. Thus the actual functional impairment of ME/CFS patients is much greater than is measured by a single CPET.

**Table 3:** **Change from Test 1 to Test 2 in functional impairment classification using VO**_**2**_**peak and ventilatory threshold criteria**[34]

**VO**_**2**_**peak(ml**^**.**^**kg**^**-1.**^**min**^**-1**^**)**	**Number of classes decreased (# participants)**	
**A** (>20)	-1 class (N = 5)*	
**B** (16–20)	-1 (2)	-2 (1)
**C** (10–15)		
**D** (<10)		
**Ventilatory threshold(ml**^**.**^**kg**^**-1.**^**min**^**-1**^**)**		
**A** (>14)	-1 (2)	-2 (2)
**B** (12–14)	-1 (3)	-2 (1)
**C** (9–10)	-1 (4)	
**D** (6–7)		
Number of participants whose impairment classification decreased on Test 2 based on criteria of VO_2_peak, VT, or both VO_2_peak and VT		
**VO**_**2**_**peak only**	**VT only**	**VO**_**2**_**peak & VT**
4	7	5

*For 5 participants, impairment classification based on Test 1 VO_2_peak decreased from “A” to “B” using Test 2 VO_2_peak.

**A** – Little-no impairment.

**B** – Mild-moderate impairment.

**C** – Moderate-severe impairment.

**D** – Severe impairment.

## Discussion

In this study, we sought to clarify the reproducibility of VO_2_ at maximal effort (VO_2_peak) and VO_2_ at ventilatory threshold (VO_2_@VT), an analog for anaerobic threshold, in patients ill with ME/CFS. Three studies to date [13,17,18] have demonstrated an abnormal post-exertional response to exercise in ME/CFS, but they do not agree as to which physiological measures fail to respond normally in ME/CFS. Second, we wanted to find out how a compromised test-retest response to exercise would impact a standard classification of functional impairment based on VO_2_peak or VO_2_@VT. Classification described by Weber and Janicki [34] was initially devised to categorize functional impairment/exercise intolerance in patients with chronic cardiac failure, although it is useful for other patient populations in which impaired gas exchange (oxygen consumption, carbon dioxide production, minute ventilation) contributes to exercise intolerance and limits physical function.

The test-retest changes in VO_2_peak that we observed are consistent with decrements reported in the three previous studies of two-day CPET response in ME/CFS [13,17,18], although the magnitude of decrease in VO_2_peak varied among these studies. In the first report to quantify an abnormal post-exertional response to exercise in ME/CFS, VanNess et al. [13] assessed the contribution of VO_2_peak measured in six females with ME/CFS and six inactive female controls to discriminate between groups. An index of maximum effort (e.g., RER) was not reported with this initial pilot study. Results indicated that a VO_2_peak decrement in test 2 alone correctly identified 6 of 6 ME/CFS and 5 of 6 controls, for an overall classification accuracy of 91.7%. Based on their reported mean data, VO_2_peak decreased during test 2 by ~22% (P = .03), in contrast to a smaller test-retest decrease observed herein of 13.8% (P < 0.001). The more robust sample size in our study may have contributed to the smaller decrease in the test-retest measures of VO_2_peak; however, for both studies, the test-retest decrement is considerably greater than <6-7% variability reported consistently in healthy subjects [19,20,23] and various patient populations [25-27,29,35-38].

A more recent two-day CPET assessment of ME/CFS by the same group [17] included 51 females with ME/CFS and 10 healthy, inactive controls. This study included measures at ventilatory threshold in a discriminant function analysis. Similar to their earlier study, CPET measures distinguished 95.1% of ME/CFS patients from healthy controls, with a cross-validation accuracy of 90.2%. The primary and secondary discriminating variables in this study were: 1) work at ventilatory threshold intensity (decreased ~55%) and 2) work at maximal intensity (decreased ~7%), respectively. In contrast to their first study [13], VO_2_peak did not contribute to the ability to distinguish ME/CFS patients in this cohort. Further, univariate analysis of VO_2_peak revealed no significant difference between test 1 and test 2 for ME/CFS, which was within normal variation for VO_2_peak.

Our results also differ from those of Vermeulen et al. [18], who measured VO_2_peak in 15 females with ME/CFS and 15 healthy female controls who were comparable in age and BMI. While there was a 2.2% increase (P < 0.05) in VO_2_peak controls, they observed a ~6.3% decrease in VO_2_peak (P < 0.01) in ME/CFS patients which is comparable to normal test-retest variation in healthy subjects. It is possible that methodological differences between their study and that of VanNess et al. [13] and our study contributed to the smaller decrease in VO_2_peak in ME/CFS patients that they detected. The cycle test protocol used by Vermeulen et al. [18] was not described in detail and appeared to vary between subjects. Reproducibility of gas exchange measures in healthy and other patient populations relies on consistent testing methodology [22]. Presumably, the protocol used for the same subject did not vary between tests, although that was not stated explicitly. Additionally, authors stated that maximum effort was assessed using RER, but the RER criterion (ie., RER ≥ 1.1) was not stated, and RER values were not reported. This is an important measure to indicate magnitude of effort, without which it is questionable whether patients gave maximal effort on both CPETs.

In addition to a 13.8% decrease in VO_2_peak in ME/CFS patients, we also observed decreases in maximal work (12.5%) and maximal heart rate (9 bpm). Likewise, Snell et al. [17] reported a decrease in maximal work of 7%. In repeat tests of leg extension strength and endurance, Paul et al. [39] also demonstrated a delayed recovery in ME/CFS work output with a greater decrease in quadriceps extension strength and endurance compared to controls following a 24 h repeat test. Conversely, Vermeulen et al. [18] reported no significant test-retest difference in maximal heart rate or work in ME/CFS subjects.

We observed a statistically significant test-retest decrease in maximal O_2_pulse of 8.8%, indicating compromised oxygen delivery in ME/CFS patients following induction of post-exertional malaise. O_2_pulse, a surrogate measure for stroke volume and arterio-venous oxygen content difference (a-vDO_2_), is a predictor of mortality in patients with cardiovascular disease [40]. It is an important index of heart function [41] and may also be associated with the onset of exercise-induced ischemia [42,43], but is also a stable and reproducible measure over time in young athletes [44] as well as adult non-athletes [45]. Vermeulen et al. [18] found a non-significant decrease of ~5% in maximal O_2_pulse in ME/CFS patients [18]. When this group later measured cardiac output and O_2_pulse during a single CPET in 178 ME/CFS patients, lower values were found in ME/CFS at VT and maximal intensities, but not at rest, compared to 11 sedentary controls. Additionally, they reported a lower arterio-venous oxygen content difference, determined non-invasively based on VO_2_ and cardiac output, and attributed these findings to lower O_2_ extraction by muscles during exercise in ME/CFS [46]. While it is not known how alteration in oxygen delivery/utilization occurs during a subsequent CPET in ME/CFS patients, these results and others [47] also suggest that the decrease in maximal O_2_pulse may partly explain the concomitant reduction in maximal workload in ME/CFS that we observed.

Our data showed a substantial decrease of 15.8% in test-retest VO_2_ at VT. Large decreases in VO_2_ at VT were also reported by VanNess et al. [13] (~27%) and Snell et al. [17] (~11%). Although the test-retest decrease (7%) reported by Vermeulen et al. [18] was not statistically significant, there was a significant group by test interaction (P < 0.05) due to an increase in control subjects. In contrast, gas exchange variables and work at VT are reliable and reproducible in healthy subjects and athletes [21,48], including test-retest differences of 1.5% for VO_2_ (r = .82-.97, Standard Error of Measurement (SE_*m*_) = 2.64 ml^.^kg^.^min^-1^), and 1.5% for cycle work (SE_*m*_ = 4.5 W) or treadmill velocity (SE_*m*_ = 10 m^.^min^-1^) (r = .95-.99). Oxygen consumption at VT in cardiac patients (Weber class A, B, C) is also stable and reproducible in multiple measures over months, albeit with somewhat more variability (CV = 9.2%) compared to healthy subjects [38].

Work measured at VT decreased 21.3% in our subjects as well as a remarkable 55% reported by Snell et al. [17]. VanNess et al. [13] did not report work at VT, and Vermeulen et al. [18] found no significant difference in the univariate comparison of test-retest work at VT, but did find a significant group by test interaction (P < 0.05). O_2_ pulse at VT decreased significantly in our subjects (12.6%) and in Vermeulen et al. [18] (9%) and was not reported by VanNess et al. [13] or Snell et al. [17].

Changes in physiological measures indicate a substantial post-exertional decrement in performance at ventilatory threshold in ME/CFS 24 hours after an initial CPET. Ventilatory or anaerobic threshold intensity indicates the workload, heart rate and/or oxygen consumption at which anaerobic metabolism begins to predominate. Thus, after induction of post-exertional malaise, the threshold lowers at which anaerobic metabolism accelerates in ME/CFS. This causes premature anaerobiosis in ME/CFS patients after they have endured an earlier physical challenge, further reducing the ability to do work. It is therefore not surprising that Snell et al. [17] found that work at ventilatory threshold contributed most substantially to differentiate ME/CFS from healthy controls.

Use of a single CPET only to indicate functional impairment in ME/CFS is problematic. The results of this study, and the consensus of the three previous studies of test-retest CPETs in ME-CFS patients, provide strong evidence of impaired physiological responses to exercise. More specifically, the abnormal post-exertional responses to exercise in ME/CFS are marked by test-retest decreases in VO_2_ and work at both maximum and ventilatory threshold intensities. Data from a single CPET resulted in classification of 12 of 22 patients as having little or no impairment, and eight as having mild/moderate impairment. Such individuals would likely be prescribed graded exercise therapy (GET) to improve aerobic capacity. However, data from the second CPET in this and prior studies [13,17,18] indicate that aerobic energy-producing processes fail to respond normally to exercise stress in ME/CFS patients. Thus, incautiously applied GET is likely to result in exacerbation of fatigue and other symptoms of ME/CFS patients.

Little is understood about the anomalous post-exertional response to exercise in ME/CFS. We know that our data does not result from any methodological or equipment problems, because during the same time period the ME/CFS patients were being tested, we performed several repeat CPETs on healthy individuals, who demonstrated comparable or better consistency and reproducibility for VO_2_peak compared to published values [19-21,23,48]. The consistently high RER values during CPET 2 provide sound evidence that ME/CFS patients can, in fact, work to maximal effort in a repeat CPET. Values for maximal RER of 1.17 and 1.14 that were reported in this study would be taken as an indication of strong, maximal efforts if reported in healthy subjects and athletes [49,50]. ME/CFS patients currently represent a unique class of ill patients who do not reproduce maximal CPET measures, unlike individuals with cardiovascular disease [27,30] lung disease [28], end-stage renal disease [26], pulmonary arterial hypertension [25] and cystic fibrosis [29].

A limitation of this study should be addressed in follow-up research. Together with the three previous studies of the two-day CPET protocol [13,17,18], the collective results demonstrate consistently abnormal CPET results in ME/CFS during test 2. However, the variation in abnormal CPET responses among these studies was not clarified in the present study and requires a larger sample size with robust statistical power.

Subsequent research should strive to address the following questions regarding post-exertional fatigue in ME/CFS. Inclusion of additional males in subsequent research should allow us to ascertain whether there are sex differences in response to the two-day CPET protocol. A large sample size will be needed to determine whether we can sub-classify ME/CFS patients based on differential responses to the two-day CPET protocol at maximal and ventilatory threshold intensities. With additional participants, it would be possible to identify clinically relevant exercise measurement cutpoints and odds ratios for use by practitioners in the diagnosis and treatment of those with ME/CFS. Physical activity prior to and following the two-day CPET should be quantified to correlate changes with the decrement measured during testing.

## Conclusions

The results of this study confirm previous work [13,17,18] that demonstrated an abnormal response to exercise in fatigued ME/CFS patients. The use of a two-day CPET protocol to measure the post-exertional response to exercise in ME/CFS allows us to better study the nature of this unusual, debilitating type of symptom exacerbation that follows exertion or stress, often described as post-exertional malaise or neuro-immune fatigue. Additionally, this test protocol yields information that can provide specific guidelines for exertion in ME/CFS patients in order to avoid symptom flares and that may improve daily physical function. ME/CFS patients exhibited significant post-exertional declines in VO_2_, work, minute ventilation and O_2_ pulse at both maximal and ventilatory threshold intensities. Consequently, classification of functional impairment based on VO_2_peak and VO_2_ at ventilatory threshold over-estimated the functional ability of 50% of ME/CFS in this sample when based on only one CPET.

## Abbreviations

BP: Blood pressure; CDC: Centers for disease control and prevention; ME/CFS: Myalgic encephalomyelitis/chronic fatigue syndrome; CPET: Cardiopulmonary exercise test; ECG: Electrocardiogram; HRpeak: Maximal heart rate; ANOVA: Analysis of variance; PEM: Post-exertional malaise; RER: Respiratory exchange ratio; RPE: Rating of perceived exertion; Ve peak: Maximal minute ventilation; VO2peak: Maximal oxygen consumption; VT: Ventilatory threshold; Work@peak: Maximal workload; O2pulse: oxygen pulse.

## Competing interests

The authors declare that they have no competing interests.

## Authors’ contributions

The funders had no role in study design, data collection and analysis, decision to publish, or preparation of the manuscript. BK wrote the first draft, and LG and JP contributed to revisions until the final manuscript was achieved. JP also contributed to collection of data and preparation of the manuscript for submission. LG contributed to data analysis and preparation of the manuscript for submission. All authors read and approved the final manuscript.
